# Transketolase-like 1 ectopic expression is associated with DNA hypomethylation and induces the Warburg effect in melanoma cells

**DOI:** 10.1186/s12885-016-2185-5

**Published:** 2016-02-22

**Authors:** Aparna Jayachandran, Pu-Han Lo, Anderly C. Chueh, Prashanth Prithviraj, Ramyar Molania, Mercedes Davalos-Salas, Matthew Anaka, Marzena Walkiewicz, Jonathan Cebon, Andreas Behren

**Affiliations:** Ludwig Institute for Cancer Research, Melbourne-Austin Branch, Heidelberg, VIC 3084 Australia; Olivia Newton-John Cancer Research Institute, Heidelberg, VIC 3084 Australia; Department of Medicine, University of Melbourne, Melbourne, VIC 3010 Australia; School of Cancer Medicine, Latrobe University, Melbourne, VIC 3086 Australia; ACRF Chemical Biology Division, The Walter and Eliza Hall Institute of Medical Research, Parkville, Melbourne, 3052 Australia; Department of Medical Biology, University of Melbourne, Parkville, Melbourne, 3010 Australia; Cancer Immuno-biology Laboratory, Olivia Newton-John Cancer Research Institute, Level 5, Olivia Newton-John Cancer and Wellness Centre, 145 Studley Road, Heidelberg, VIC 3084 Australia; School of Medicine and the Gallipoli Medical Research Foundation, The University of Queensland, Brisbane, QLD 4120 Australia

**Keywords:** *TKTL1*, Warburg effect, Aerobic glycolysis, Melanoma, Hypomethylation

## Abstract

**Background:**

The metabolism of cancer cells is often reprogrammed by dysregulation of metabolic enzymes. Transketolase-like 1 (TKTL1) is a homodimeric transketolase linking the pentose-phosphate pathway with the glycolytic pathway. It is generally silenced at a transcriptional level in somatic tissues. However, in human cancers its expression is associated with the acquisition of a glycolytic phenotype (the Warburg effect) by cancer cells that contributes to the progression of malignant tumors. In melanoma, defective promoter methylation results in the expression of genes and their products that can affect the tumor cell’s phenotype including the modification of immune and functional characteristics. The present study evaluates the role of *TKTL1* as a mediator of disease progression in melanoma associated with a defective methylation phenotype.

**Methods:**

The expression of TKTL1 in metastatic melanoma tumors and cell lines was analysed by qRT-PCR and immunohistochemistry. The promoter methylation status of *TKTL1* in melanoma cells was evaluated by quantitative methylation specific PCR. Using qRT-PCR, the effect of a DNA demethylating agent 5-aza-2’-deoxycytidine (5aza) on the expression of *TKTL1* was examined. Biochemical and molecular analyses such as glucose consumption, lactate production, invasion, proliferation and cell cycle progression together with ectopic expression and siRNA mediated knockdown were used to investigate the role of *TKTL1* in melanoma cells.

**Results:**

Expression of *TKTL1* was highly restricted in normal adult tissues and was overexpressed in a subset of metastatic melanoma tumors and derived cell lines. The *TKTL1* promoter was activated by hypomethylation and treatment with 5aza induced *TKTL1* expression in melanoma cells. Augmented expression of *TKTL1* in melanoma cells was associated with a glycolytic phenotype. Loss and gain of function studies revealed that *TKTL1* contributed to enhanced invasion of melanoma cells.

**Conclusions:**

Our data provide evidence for an important role of *TKTL1* in aerobic glycolysis and tumor promotion in melanoma that may result from defective promoter methylation. This epigenetic change may enable the natural selection of tumor cells with a metabolic phenotype and thereby provide a potential therapeutic target for a subset of melanoma tumors with elevated *TKTL1* expression.

**Electronic supplementary material:**

The online version of this article (doi:10.1186/s12885-016-2185-5) contains supplementary material, which is available to authorized users.

## Background

Melanoma is a cancer derived from neuroectoderm that is often fatal once it has metastasised [1, 2]. Current treatment approaches include the molecular targeting of oncogenic gene products and immune-based therapies, both of which induce significant responses in many patients with advanced disease [3, 4]. Despite this, many patients develop therapy resistance or do not respond to treatment [5–7]. Since alterations in cellular metabolism may contribute to the malignant phenotype, targeting the metabolic circuitry of melanoma cells may offer a promising additional therapeutic strategy [8, 9].

The reprogramming of cellular metabolism is one of the hallmarks of cancer [10]. Whereas normal cells direct glucose to mitochondrial oxidative phosphorylation to generate ATP when oxygen is abundant, cancer cells generally exhibit greater glucose uptake and lactate secretion, regardless of oxygen availability. This phenomenon is termed “aerobic glycolysis” or the “Warburg effect” [10–12]. This can benefit cancer cells by facilitating increased proliferation, enhanced invasion and resistance to apoptosis, which in turn promote tumor progression and metastasis [13]. These observations have raised the possibility that targeting metabolic pathways that the cancer cell depends upon may be a useful therapeutic strategy. Metabolic profiling of melanoma cells has revealed their dependence upon the Warburg effect as the major source of energy [14]. Moreover, this phenomenon is the basis for ^18^FDG-PET (Fluorodeoxy glucose positron emission tomography) to image the metabolic activity of cancer in patients [15].

Transketolases are essential and rate-limiting enzymes in the non-oxidative part of the pentose-phosphate pathway [16, 17]. An augmented pentose-phosphate pathway in some tumors enables oxygen-independent glucose conversion to ribose for nucleic acid synthesis, glucose degradation to lactate, and regeneration of redox equivalents [18]. The transketolase family includes Transketolase (TKT), and two Transketolase-like proteins (TKTL1 and TKTL2) [19]. Among them, TKTL1 is the isoform specifically upregulated in different human cancers such as head and neck, lung, breast, stomach, colon, nephroblastoma and endometrial cancer [20–26]. Its overexpression predicts poor patient survival, tumor recurrence and resistance to chemo and radiation therapy in many cancers [23, 27, 28]. TKTL1 expression was demonstrated in invasive tumors which correlated with increased metastasis [29]. Some of the tumor types expressing TKTL1 have responded to the transketolase inhibitor Oxythiamine that effectively blocked cell proliferation [30, 31]. TKTL1 has recently been used as a biomarker in a blood test based on the epitope detection in monocytes (EDIM) technology allowing for the non-invasive detection of neoplasia and tumor recurrence [32]. Although TKTL1 expression in conjunctival melanoma tumors has been reported, the mechanism by which expression becomes unregulated and the functional consequences of aberrant TKTL1 expression in melanoma remains unclear and these warrant further investigation [33].

Methylation defects are commonly observed in melanoma and this can result in the expression of gene products that are otherwise silenced at a transcriptional level in somatic tissues. This can profoundly affect the phenotype of the cancer cell. Among these re-expressed protein are the Cancer Testis or Cancer Germ-line family of antigens (CTAg); so called because they are often immunogenic and can mediate the immune rejection of cancer [34]. They are associated with disease progression and may be associated with poorer clinical outcomes in melanoma and other cancers [35, 36]. Many of these are coded on the X chromosome and their function is poorly understood although the association with stem-like characteristics has been reported [37]. Given that *TKTL1* is also an X chromosome coded molecule (Xq28) that, like the CTAg, is generally repressed in somatic tissues, we sought to determine if DNA hypomethylation also induced aberrant expression of *TKTL1* in melanoma and to assess its role in promoting the Warburg effect in melanoma cells.

We detected increased expression of TKTL1 in a subset of metastatic melanoma tumors and cell lines and found TKTL1 expression in melanoma tumors was associated with promoter hypomethylation. We demonstrated that the *TKTL1* promoter could be activated by treatment with 5-aza-2’-deoxycytidine (5aza) thereby inducing *TKTL1* expression in melanoma cells. Elevated TKTL1 expression enhanced the Warburg effect by accelerating glucose utilisation and lactate production and TKTL1 loss and gain of function studies revealed that TKTL1 enhanced invasion in melanoma cells. Taken together, our data suggests that a subset of melanomas with defective methylation rely on TKTL1-dependent aerobic glycolysis and have enhanced tumorigenesis. These may be amenable to inhibition of the Warburg effect by therapies that target TKTL1.

## Results

### A subset of metastatic melanoma tumors and cell lines express TKTL1

**Fig. 1:**
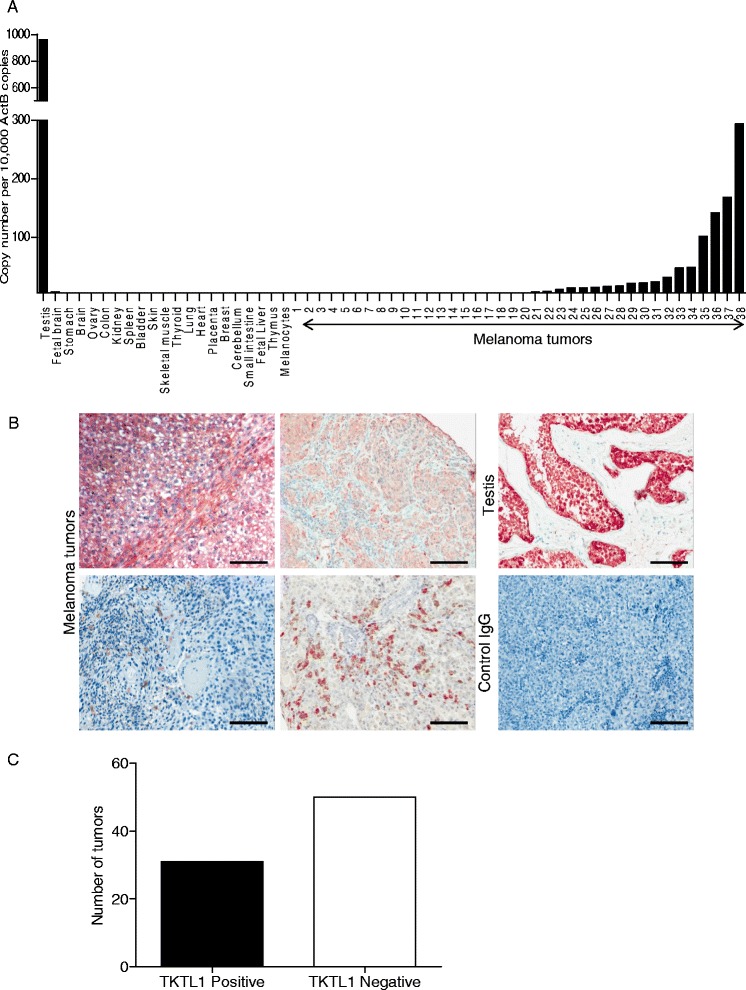
TKTL1 is highly expressed in human testis and melanoma tumors. **a** qRT-PCR was employed to measure the expression of *TKTL1* in a panel of normal human tissues and in 38 metastatic melanoma tumor samples. **b** TKTL1 immunohistochemical staining in testis as positive control and control IgG staining in tumors as negative control are demonstrated. Representative staining patterns for TKTL1 in metastatic melanoma tumors are shown. Original magnification, 10 ×. **c** Graph shows number of *TKTL1* positive and negative tumors

We quantified *TKTL1* transcripts by qRT-PCR in a panel of normal human tissues and metastatic melanoma tumors. Figure 1a shows that high expression of *TKTL1* mRNA was detected in testis but no other normal human tissues tested including skin and melanocytes. 15 of 38 (40 %) melanoma tumors assessed expressed *TKTL1* to varying degrees. We examined TKTL1 by immunohistochemistry using a tissue microarray (TMA) comprising 81 tumors from patients with stage III and IV metastatic melanoma. Four representative tumors with high and diffuse intensity staining of tumor cells for TKTL1 are depicted in Fig. 1b. TKTL1 in tumors varied from homogenous to heterogeneous expression with clusters of TKTL1 positive cells interspersed with TKTL1 negative cells. TKTL1 expression in testis tissue was used as positive control and anti-IgG antibody was used as negative control (Fig. 1b). 31 of 81 (38 %) of metastatic melanoma tumors were scored positive for TKTL1 expression (Fig. 1c). High magnification image shows that TKTL1 localization is predominantly cytosolic and membrane staining is also seen in some melanoma tumor cells. Nuclear staining in some tumors is Melanin (brown) and not TKTL1 (red) (Additional file 1: Figure S1A). We extended our studies to a clinical outcome dataset that subdivided 57 stage IV melanoma samples into high-risk or low-risk melanoma groups based on transcriptome profiling (GSE22153) [38]. We found that tumors expressing high *TKTL1* levels were statistically significantly associated with the high-risk group (log-rank p value 0.0277), risk being reduced survival and risk of relapse.

**Fig. 2:**
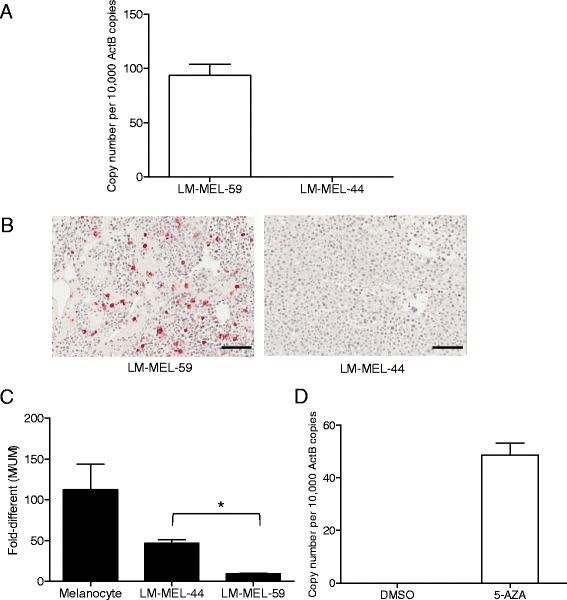
TKTL1 is expressed in metastatic melanoma cell lines and is regulated by promoter hypomethylation. **a** Using qRT-PCR the expression of *TKTL1* in two melanoma cell lines, LM-MEL-59 and LM-MEL-44 was determined. **b** TKTL1 expression in LM-MEL-59 and LM-MEL-44 was determined by IHC. Original magnification, 20×. **c** Bisulfite conversion and MS-qPCR was performed to assay the promoter methylation status of *TKTL1* in melanocytes and metastatic melanoma cells. Relative levels of methylated and unmethylated products (M/UM) was quantified. **d** QRT-PCR was employed to detect changes in expression of *TKTL1* by 5-azacytidine treatment in LM-MEL-44 cell line. Values are ± SD of three independent experiments in triplicate (*, *p* < 0.05)

In a panel of 53 metastatic melanoma cell lines *TKTL1* mRNA was detectable in 11 of 53 lines (20 %) by qRT-PCR (Additional file 1: Figure S1B). We selected two cell lines for further functional studies; LM-MEL-59, which showed the highest level of *TKTL1* mRNA expression and LM-MEL-44 which lacked *TKTL1* expression (Fig. 2a). Immuno-staining confirmed the expression of TKTL1 in LM-MEL-59 and its absence in LM-MEL-44 (Fig. 2b). Both of these cell lines lack activating mutations in the BRAF protein kinase. High magnification image confirms cytosolic TKTL1 localization (Additional file 1: Figure S1C).

### Promoter hypomethylation of the *TKTL1* gene is responsible for its activation in melanoma

Expression of *TKTL1* has been reported to be controlled by methylation and demethylation of *TKTL1* has been previously reported in lung and head and neck cancers [24, 39]. However, little is known about the methylation status of *TKTL1* in melanoma. To investigate promoter cytosine-(phosphate)-guanine (CpG) dinucleotide DNA methylation, we quantified the level of *TKTL1* promoter methylation using bisulfite modification and MS-qPCR. Melanocytes were found to be hypermethylated which correlated with the lack of *TKTL1* expression in those cells (Fig. 2c), while metastatic melanoma cell lines tested showed reduced methylation levels when compared to melanocytes. LM-MEL-44 cell line with no *TKTL1* expression showed hypermethylation of the *TKTL1* promoter whereas a lesser degree of *TKTL1* promoter methylation was detected in LM-MEL-59 with high *TKTL1* mRNA expression. Promoter hypermethylation of *TKTL1* could be reversed by a DNA demethylating agent 5-aza-2′-deoxycytidine (5aza) in LM-MEL-44 resulting in the expression of *TKTL1* in treated LM-MEL-44 cells in contrast to DMSO treated cells (Fig. 2d).

We extended our studies to a publically available clinical melanoma patient dataset to determine whether deregulation of *TKTL1* expression was linked to DNA hypomethylation. Using MethPrimer (www.urogene.org/cgi-bin/methprimer/methprimer.cgi), a CpG island prediction program, we identified two CpG sites in the *TKTL1* promoter that were highly prone to methylation (data not shown). The primers we designed for MS-qPCR spanned these two CpG sites; cg09892236 (−100 bp of transcription start site (TSS)) and cg23106779 (−250 bp of TSS). We used an in silico approach to assess the methylation status of the promoter region on the *TKTL1* gene in 385 metastatic melanoma tissues from the Skin Cutaneous Melanoma (SKCM) dataset of The Cancer Genome Atlas (TCGA) database (www.cbioportal.org) [40, 41] and estimated its association with *TKTL1* mRNA expression. We first evaluated DNA methylation of the two CpG sites using the Illumina Infinium Human Methylation 450 k BeadChip array (450 k array). Methylation of the two CpG sites were statistically associated with each other with the melanoma dataset (correlation = 0.68, p-value = 2.2e-16). We next examined the exon and gene expression pattern of *TKTL1* in these patient samples in the Illumina Hiseq RNA-Seq Platforms. Spearman correlation coefficients were calculated to test the correlations between DNA methylation and gene expression of *TKTL1*. We found that methylation status at both the CpG sites was statistically inversely correlated with *TKTL1* gene expression in melanoma samples (Table 1 and Additional file 2: Figure S2A&B). Melanoma samples were further sub-divided into two groups, primary (*n* = 82) and metastatic (*n* = 303) in order to examine the role of DNA methylation in regulation of *TKTL1* gene expression within primary and metastatic melanoma samples. We found positive correlation of *TKTL1* expression with methylation in metastatic samples (Table 2). These results confirm that DNA hypomethylation is associated with aberrant activation of *TKTL1* expression in metastatic melanoma.

**Table 1:** Integrative analysis of methylation and gene expression in Melanoma samples (TCGA data analysis)

Probe Id (450 k)	Methylation and gene expression (*n* = 385)		Methylation and exon expression (*n* = 385)	
	Rho	*P*-value	Rho	*P*-value
cg09892236	−0.14	0.005	−0.16	0.0024
cg23106779	−0.11	0.04	−0.13	0.01

**Table 2:** Integrative analysis of methylation and gene expression in primary and metastatic samples (TCGA data analysis)

Probe Id (450 K)	Methylation and gene expression				Methylation and exon expression			
	Primary (*n* = 82)		Metastatic (303)		Primary (82)		Metastatic (303)	
	Rho	*P*-value	Rho	*P*-value	Rho	*P*-value	Rho	*P*-value
cg09892236	−0.097	0.38	−0.15	0.006	−0.10	0.32	−0.17	0.004
cg23106779	−0.10	0.36	−0.09	0.09	−0.16	0.13	−0.11	0.05

### TKTL1 is co-expressed with Cancer Testis Antigens in melanoma tumors

The epigenetic reactivation of TKTL1 occurs simultaneously with DNA hypomethylation of CTAgs in head and neck cancer [39]. To determine whether *TKTL1* expression in melanoma was linked to the expression of CTAgs, we queried the TCGA database comprising of 261 melanoma patient samples. We found a significant tendency toward co-occurrence of many CTAgs with *TKTL1* expression in melanoma tumors (Table 3).

**Table 3:** Gene expression correlation p value for the co-occurrence of each gene pair across 261 melanoma patient tumors

Cancer Testis Antigen	Gene symbol	TKTL1 (Adjusted *p* value)
Ropporin	ROPN1	8.33E-08
Testis-specific serine kinase 6	TSSK6	0.001107
Transcription factor DP family, member 3	TFDP3	0.058881
Sperm autoantigenic protein 17	SPA17	0.061708
Armadillo repeat containing 3	ARMC3	0.061708
Testis expressed 15	TEX15	0.11398
Synovial sarcoma, X breaking point 1	SSX1	0.201966
Centrosomal protein 290 kDa	CEP290	0.254303
Synovial sarcoma, X breaking point 5	SSX5	0.280049

### Altered expression of TKTL1 affects the glycolytic metabolism of melanoma cells

**Fig. 3:**
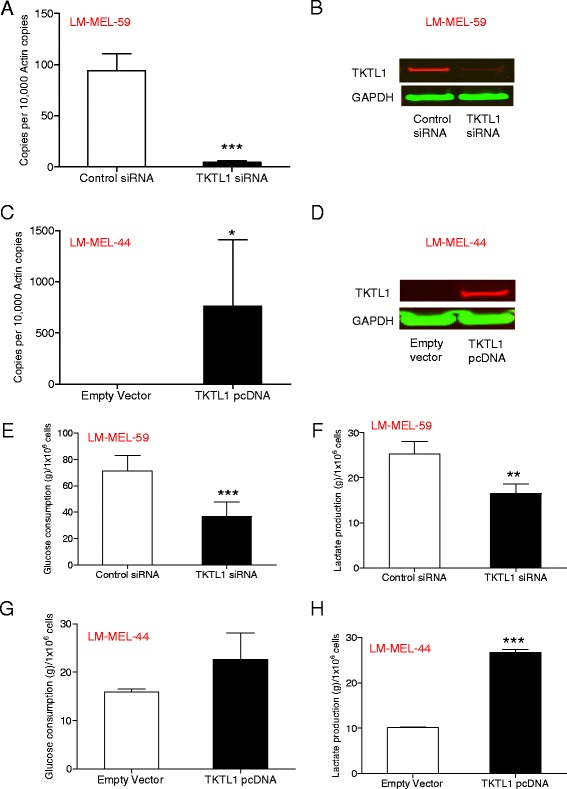
Ectopic overexpression and knockdown of TKTL1 influences the Warburg effect in melanoma cells. **a** QRT-PCR was employed to evaluate the expression of *TKTL1* in LM-MEL-59 after treatment with either *TKTL1* or control siRNA for 72 h. **b** Western Blotting with a mouse monoclonal anti-TKTL1 antibody showed reduction in TKTL1 levels after siRNA treatment in LM-MEL-59 after 72 h. GAPDH was used a loading control. **c** *TKTL1* expression was assessed by qRT-PCR in LM-MEL-44 cells transfected with *TKTL1* pcDNA or empty control for 72 h. **d** Immunoblot of TKTL1 confirmed expression of TKTL1 after transfection of a *TKTL1* expression vector in LM-MEL-44. Blots were probed with GAPDH as a control for loading and transfer. Glucose consumption was measured in cell free supernatant of **e** LM-MEL-59 cell line following treatment with *TKTL1* or control siRNA for 72 h and **g** LM-MEL-44 cell line following over-expression of *TKTL1* or empty vector control for 72 h. The production of lactate in culture supernatants was measured in **f** LM-MEL-59 after knockdown of *TKTL1* for 72 h and **h** LM-MEL-44 after treatment with *TKTL1* pcDNA or empty vector control for 72 h. Values are ± SD of three experiments in triplicate (**, *p* < 0.005, ***, *p* < 0.0005)

To determine the role of TKTL1 in melanoma, we sought to manipulate TKTL1 levels in melanoma cell lines by siRNA-mediated knockdown and vector-mediated over-expression. Effective suppression of TKTL1 on mRNA and protein levels in LM-MEL-59 was achieved at 10 nM concentration of siRNA (Fig. 3a & b). To confirm that the effects shown by TKTL1 were not off-target effects, we performed microarray [42] and qRT-PCR for the isoforms of *TKTL1* namely, *TKT* and *TKTL2*. We found that *TKT* levels were high in both LM-MEL-59 and LM-MEL-44, while *TKTL2* levels were low. Additionally, we have used testis tissue as a positive control for the expression of these genes. Upon knockdown with *TKTL1* siRNA, *TKT* levels were affected by one of the siRNAs but only modestly by the other (Additional file 3: Figure S3).

Transfection with a *TKTL1* expression plasmid resulted in marked overexpression of TKTL1 in LM-MEL-44, demonstrated by qRT-PCR and immunoblotting (Fig. 3c & d). No additional bands were detected for TKTL1 as evident in Additional file 4: Figure S4A&B. High transfection efficiency was also confirmed with immunofluorescence staining (Additional file 4: Figure S4C). Elevated glucose uptake and lactate production have been detected during malignant transformation. These metabolic parameters of cancer cells are consistent with a pronounced glycolytic state [43, 44]. To investigate whether TKTL1 expression in melanoma was driving the Warburg effect, glucose consumption and lactate production were assayed after TKTL1 interference. Measurements were made on conditioned medium of LM-MEL-59 cells after treatment with *TKTL1* or control siRNA for 72 h. Depletion of *TKTL1* in these cells caused a substantial decrease in glucose consumption and associated reduction in lactate production (Fig. 3e & f) while ectopic expression of TKTL1 in LM-MEL-44 conferred a more glycolytic phenotype (Fig. 3 g & h). Collectively, these results showed that TKTL1 enhanced aerobic glycolysis in melanoma.

### TKTL1 affects proliferation in melanoma cells

**Fig. 4:**
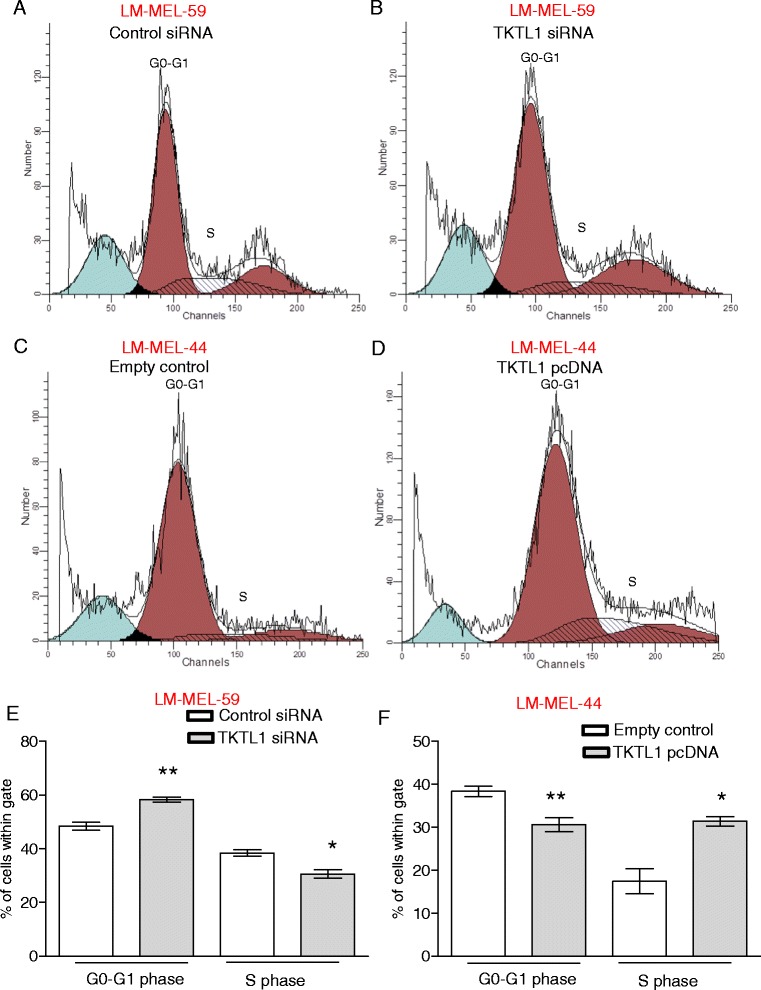
Loss of TKTL1 expression changes cell cycle distribution of melanoma cells. Cell cycle phases were determined by propidium iodide staining of melanoma cells and subsequent flow cytometric analysis. A representative histogram of cell cycle analysis of LM-MEL-59 is shown after **a** control siRNA treatment and **b** *TKTL1* siRNA treatment. Analysis of percentage of cells in **e** G0-G1 phase and S phase cell after treatment of LM-MEL-59 with *TKTL1* or control siRNA. Values are ± SD of three experiments in triplicate (*, *p* < 0.05, **, *p* < 0.005). Histograms depicting distribution of cell cycle phase in LM-MEL-44 after treatment with **c** empty vector control and **d** *TKTL1* pcDNA. Cell cycle distribution of **f** GO-G1 and S phase cell population after 48 h of ectopic expression of TKTL1 or empty vector in LM-MEL-44 was performed. Values are ± SD of three experiments in triplicate (*, *p* < 0.05, **, *p* < 0.005)

TKTL1 plays an important role in tumor proliferation in gastric, colon and hepatocellular carcinomas [19, 26, 45]. We investigated whether TKTL1 plays a similar role in melanoma. *TKTL1* knockdown in LM-MEL-59 led to an increase in the percentage of G0-G1 phase cells and a decrease in the percentage of S phase cell population compared to control transfected cells (Fig. 4a, b & e) as demonstrated by cell cycle analysis. Using MTS assay to determine total cell number after *TKTL1* knockdown, similar changes were detected. However, effects were only modest after 48 h (Additional file 5: Figure S5A).

Conversely, *TKTL1* overexpression led to a decrease in the percentage of G0-G1 phase cells in LM-MEL-44 and an increase in the percentage of S phase cells (Fig. 4c, d & f). Table 4 depicts the cell cycle phase distributions under the different conditions. Similarly, MTS assay demonstrated slightly enhanced proliferation in TKTL1 overexpressing cells compared to empty vector treated cells, although no significance was achieved (Additional file 5: Figure S5B). Overall, these results indicate that TKTL1 levels may affect melanoma cell proliferation.

**Table 4:** Comparison of cell cycle distribution after TKTL1 knockdown and overexpression

Treatment (LM-MEL-59)	Sub G1 (%)	G0-G1 (%)	S (%)	G2-M (%)
Control siRNA	8.27	48.26	38.84	12.89
*TKTL1* siRNA	7.9	58.17	30.71	11.11
*p* Value	0.78	0.004	0.01	0.65
Treatment (LM-MEL-44)	Sub G1 (%)	G0-G1 (%)	S (%)	G2-M (%)
Empty vector control	18.05	79.55	17.39	3.05
*TKTL1* pcDNA	8.89	65.55	31.42	3.05
*p* Value	0.02	0.001	0.01	0.99

### TKTL1 promotes invasion in melanoma cells

**Fig. 5:**
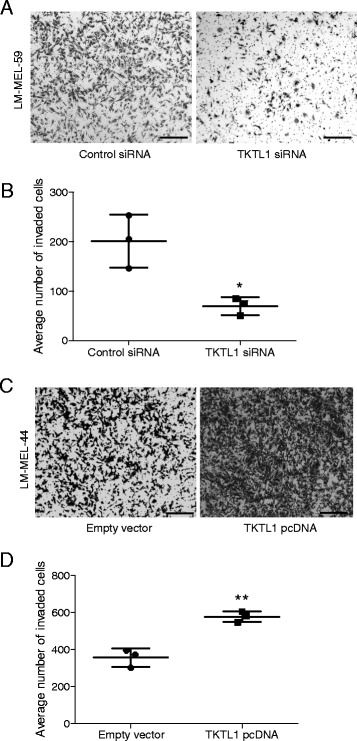
TKTL1 enhances invasive behaviour in melanoma. Melanoma cells were plated out and transfected with either 10nM control siRNA or *TKTL1* specific siRNA and subjected to invasion assay. **a** Representative images of invasion of LM-MEL-59 is shown (scale bar = 100 μm). **b** The graph shows the total number of invasive cells counted. **c** Invasiveness of melanoma cells LM-MEL-44 after treatment with empty vector control or *TKTL1* pcDNA was tested, images captured (scale bar = 100 μm) and **d** invasion was quantified as above. Values are mean ± SD of three independent experiments in triplicate (*p < 0.05, **p < 0.005)

Next, we sought to investigate the effect of TKTL1 on cell invasion. *TKTL1* depleted LM-MEL-59 cells were significantly less invasive, while *TKTL1* overexpressing LM-MEL-44 cells were more invasive than controls in the matrigel-coated Boyden chamber assay (Fig. 5a & c). *TKTL1* depleted cells showed 62 % loss and *TKTL1* overexpressing cells showed 38 % increase in invasion (Fig. 5b & d). These results demonstrate a substantive effect of TKTL1 levels on melanoma cellular invasion.

### Oxythiamine does not inhibit proliferation and invasion in melanoma cells

In tumors expressing high TKTL1, oxythiamine has been used to block proliferation effectively both *in vitro* and *in vivo* [46]. We treated both LM-MEL-59 and LM-MEL-44 with the inhibitor in varying dose of 10 and 100 μM (Additional file 6: Figure S6A&B). In both cell lines no reduction in proliferation was detected. To assess if Oxythiamine induced a G1 phase cell cycle arrest, LM-MEL-59 cells treated for 72 h with 100 μM of the inhibitor was subjected to flow cytometric cell cycle analysis. However, Oxythiamine treatment in our hands did not cause a G1 phase arrest in melanoma cells (Additional file 6: Figure S6C). Furthermore, Oxythiamine treatment did not alter invasive potential (Additional file 6: Figure S6D & E).

## Discussion

Recent studies have shown that TKTL1 plays an important role in the development and progression of human tumors [20–26, 29, 47, 48]. In this study, we report that TKTL1 expression was upregulated in a significant subset of metastatic melanoma tumors and cell lines. In agreement with other reports, we observed that increased TKTL1 resulted in accelerated glucose uptake and lactate secretion in melanoma cells and this was associated with changes in the proliferation and invasion in melanoma cells. Moreover, we could demonstrate that the *TKTL1* promoter is hypomethylated in melanoma cells compared with melanocytes and consequently *TKTL1* expression could be induced by incubation of melanoma cells with 5aza. We found a significant association of hypomethylation with *TKTL1* gene expression in melanoma patient samples. Additionally, to our knowledge, this is the first study evaluating the effect of TKTL1 expression on metabolic and cellular functions in melanoma.

Abnormal DNA methylation dysregulates gene expression in cancer and this has been associated with changes in phenotype and function. In melanoma X-chromosome coded molecules that can be affected include the CTAg and TKTL1. Simultaneous reactivation of TKTL1 with CTAg such as the melanoma antigen family (MAGE-A) has been reported in lung and head and neck cancers [39]. In melanoma tumors, we have identified that TKTL1 and CTAgs showed significant correlation in expression. Most of these CTAgs have been implicated in tumorigenesis [49–52]. Some of these CTAgs such as Ropporin, TSSK6, SPA17 and TEX15 have been reported to play important roles in sperm motility and knockout mice lacking these genes are often sterile due to profound impairment in sperm motility [53–56]. CTAgs are repressed by epigenetic mechanisms during development and it has been suggested that the coordinated pathologic reversal of developmental epigenetic events often occurs in cancer cells [39]. We speculate that in melanoma aberrant demethylation occurs as a common underlying mechanism that links transcriptional activation of TKTL1 and CTAgs that may cooperate to promote cancer progression by mechanisms such as Warburg effect, motility, proliferation and apoptosis. Aberrant expression of CTAgs has previously been associated with poor outcomes in melanoma and other cancers [34, 36]. It has never been clear why increased expression of these antigens should paradoxically carry a poorer prognosis. This study may provide the clue, that increased antigen expression and TKTL1 expression are both a consequence of a phenotype that results from methylation changes, and it is this phenotype that associates pro-tumorigenic metabolic changes with bystander tumor antigen expression.

Our finding that a subset of metastatic cutaneous melanoma express TKTL1 at the mRNA and protein levels is consistent with the report of Lange et al., showing that conjectival melanoma tumors expressed TKTL1 [33]. We also noted elevated levels of *TKTL1* in normal testis tissue, where aerobic glycolysis has been previously detected [57]. Although the Warburg effect appears to be a wasteful form of metabolism compared to the mitochondrial driven oxidative phosphorylation, it is a mechanism for rapid generation of energy [58].

Heterogeneity within LM-MEL-59 for TKTL1 expression is representative of most of the tumors that stained for TKTL1. Ho J et al. reported two distinct populations of cells that rely on either glycolysis or oxidative phosphorylation exist within melanoma tumors and can be metabolically linked such that lactate from the glycolytic portion of the tumor helps fuel ATP production through the oxidative phosphorylation in a process termed metabolic symbiosis [59]. It is conceivable that TKTL1 positive cells may rely solely on the Warburg effect in contrast to the TKTL1 negative cells that may rely on mitochondrial oxidative phosphorylation to support cell growth. Metabolic symbiosis of TKTL1 positive versus negative cells warrants further investigation using purified cell populations.

Recent studies have reported oxidative respiration plays an important role in melanoma metabolism in addition to glycolysis *in vitro* [14, 59, 60]. Activating mutations in the BRAF protein kinase are the most common genetic alterations in melanoma, found in around 40 % of tumors. Many studies have shown a role for BRAF signalling in the regulation of melanomas metabolism [8, 61, 62]. Melanomas treated with agents that target oncogenic BRAF undergo a reversal of the Warburg effect and become dependent on oxidative phosphorylation [61, 62]. However, both cell lines used to investigate the role of TKTL1 in this study lack the activating mutations in BRAF, so BRAF mutations do not appear to be a prerequisite for TKTL1-driven aerobic glycolysis. This is consistent with TKTL1 being activated through an epigenetic mechanism that is independent of BRAF mutational status. A glycolytic profile of melanoma cell lines has recently been identified by metabolic profiling and the study reported that melanoma cells were inherently more glycolytic than melanocytes and could adapt their metabolic program to environmental challenges and also found that all melanoma cells evaluated maintained functional mitochondria [14]. This does not invalidate a role for the Warburg effect, and suggests that metabolic flexibility may occur, enabling a survival advantage for tumors growing in diverse environments. The mechanisms that tip the balance towards more glycolysis and less respiration in tumors are emerging.

In circumstances where glucose metabolism exceeds the capacity of a cell to assimilate or store glucose-derived carbon, cells with high levels of TKTL1 expression have a growth advantage when compared to those with low levels of TKTL1. A correlation between glucose uptake and proliferation has been demonstrated in several cell lines, for instance in ovarian adenocarcinoma and breast cancer cells [63, 64]. Our finding that TKTL1 expression enhances proliferation is consistent with these findings. The important question is whether this reflects a dependence on TKTL1 as a mechanism for tumor progression or survival. If so, it may be a useful therapeutic target, in those cases where it is overexpressed.

Langbein et al. examined 70 colon cancers and found increased TKTL1 protein levels in invasive tumors compared to non-invasive tumors and healthy tissue [17]. High levels of TKTL1 were also correlated with invasion and poor prognosis in urothelial, laryngeal squamous and colorectal cancer [17, 29]. Our observation that TKTL1 promotes invasiveness *in vitro* indicates that the metabolic pathways that support tumor progression are likely to be complex. Cells with increased TKTL1 showed increased rate of glycolysis with production of high levels of lactate. Cancer cells in general seem to adapt to the acidic conditions better than surrounding cells and this may promote the degradation of host matrix leading to greater invasiveness and thereby contributing to metastases. Furthermore, the acidic microenvironment can enhance survival of cancer cells by inhibiting anti-cancer immune responses [65, 66]. Thus, the TKTL1-driven metabolic adaptation may promote cancer progression through a variety of mechanisms.

Although our results suggest that TKTL1 could be a therapeutic target in melanoma, treatment with oxythiamine, a thiamine antagonist, was ineffective even though it was previously shown to decrease proliferation in tumors with high TKTL1 expression [46]. Similarly, earlier studies demonstrated that oxythiamine was not effective in most thyroid cancer cells expressing high TKTL1 and in vivo in normal rat tissues that expressed high TKTL1 [30, 67]. Using computer modelling, 38 amino acid deletion was introduced into the normal human TKT gene to obtain a variant analogous to TKTL1. However, this variant lacked catalytic activity exhibited by the TKT protein, and Meshalkina et al. therefore suggest that TKTL1 is distinct from the thiamine diphosphate dependent enzyme TKT [68]. Our data suggests that we cannot rule out the possibility that TKT acts in concert with TKTL1 as we observed that TKT is highly expressed in melanoma cell lines and knockdown of TKTL1 impacts TKT expression, if only modestly. However, given the fact that the overexpression of TKTL1 in LM-MEL-44 showed opposing effects, and that one of the siRNAs is not changing TKT levels significantly while having the same functional effects in LM-MEL-59, TKTL1 is clearly the main player in the observed effects. Recently, more specific inhibitors of transketolases have been developed [69]. Further studies will determine whether these are more effective on TKTL1-expressing cancers.

## Conclusion

The functional significance of the increased TKTL1 in melanoma, its association with DNA hypomethylation and the Warburg effect has not previously been reported. Here, we demonstrate that melanoma cells expressing TKTL1 are predominately glycolytic and that TKTL1 expression increases both cellular proliferation and invasion. Melanoma cells exhibit metabolic plasticity and compensatory mechanisms that allow metabolic adaptations have previously made it difficult to exploit metabolic targets for therapy. Here we show that a methylation defect likely leads to TKTL1 over expression. We postulate that this overexpression allows survival within the hypoxic tumor micro-environment and selects for cells that bear it. Consequently their dependence on glycolysis may arise through a mechanism that does not reflect metabolic plasticity. This in turn points to the potential of targeting TKTL1-expressing melanomas particularly if they prove to be dependent on it for their survival. Ultimately the success of such a strategy will depend on this and on the quality of any drugs that are developed. Further investigations will determine the extent of which is useful target for therapeutic intervention in metastatic melanoma.

## Methods

### Cell culture and archival patient materials

Melanoma cell lines were established from resected melanoma metastases by mechanical dissociation of tissue with subsequent overnight digestion in media containing collagenase IV and DNAse at 37 °C. Established cell lines were Mycoplasma-tested using the MycoAlert test (Lonza Rockland, Inc.) and STR-profiled for identity confirmation.

Following pathological examination post-surgery tumour fragments were formalin-fixed and paraffin-embedded (FFPE) by staff at the Austin Health Tissue Bank. All melanoma tissue was derived from metastases resected from regions distant to the primary tumor; no locoregional metastases or primary tumors were included. Tissue microarray consists of tumor tissue embedded in a block and allows analysis of multiple patient tissue samples on the same slide. The study cohort consisted of 81 patients with stage III and IV metastatic melanoma. All tissue donors provided written informed consent for tissue collection and research, which was covered by protocols approved by the Austin Health Human Research Ethics Committee, Melbourne, Australia (approval number H2012/04446). All cell lines were matched with their donors by HLA-typing. Cells were cultured in RPMI1640 supplemented with 10 % fetal calf serum (FCS) as described previously [42].

### Quantitative reverse transcriptase-PCR (qRT-PCR)

Total RNA was extracted using the RNEasy mini kit (Qiagen). We have performed on column DNAase digestion using RNase-Free DNase at room temperature (20–30 °C) for 15 min in accordance to Qiagen RNEasy Kit instructions. Spectrophotometric quantification using Nanodrop confirmed purity of RNA and absence of DNA in our samples. In addition to this to prevent any interference by residual DNA contamination, we have performed qRT-PCR with primers that are intron spanning such that genomic DNA will not be amplified.

RNA was reverse transcribed using the High Capacity cDNA Reverse Transcription Kit (Applied Biosystems). Following reverse transcription, qRT-PCR was performed using the SensiFAST SYBR Lo-ROX kit (Bioline), and a Vii-A7 thermocycler (Applied Biosystems). The primers for *TKTL1* and *β-actin* (internal control) were synthesized by Sigma Aldrich and the sequences were as follows: *TKTL1* primer (forward) 5’-CCA CCT GAT TAC AGA GTT GGT G-3’ and (reverse) 5’-CTC TGT TGT TCG CGT AGC C-3'; *β-actin* (forward) 5'-CCA ACC GCG AGA AGA TGA-3’ and (reverse) 5'-CCA GAG GCG TAC AGG GAT AG-3’, *TKT* primer (forward) 5’-TGT GTC CAG TGC AGT AGT GG-3’ and (reverse) 5’-ACA CTT CAT ACC CGC CCT AG-3’; *TKTL2* primer (forward) 5’-ACG ACC GGT TCA TCC TCT C-3’ and (reverse) 5’-TCC ACC CAA GCA GCA TAG A-3’.

### Immunohistochemistry

Immunohistochemistry was performed on tissue microarrays constructed from formalin-fixed paraffin-embedded (FFPE) metastatic melanomas and melanoma cell lines after citrate buffer (pH 6.0) antigen retrieval for 30 min using the Dako Envision^+^ kit (Dako). Incubation with 1× protein blocking buffer for one hour at room temperature was followed by overnight incubation at 4 °C with mouse monoclonal anti-TKTL1 antibody (clone JFC12T10; 1:200 dilution) previously described by Langbein et al. [17]. 60 min incubation with secondary anti-mouse antibody HRP (Dako) was performed. 3-amino-9-ethylcarbazole (AEC) was used as chromagen (Vector Laboratories). Slides were counterstained with hematoxylin, images were captured and immunohistochemical reactivity was evaluated by two independent investigators. Patient tumor cores were scored TKTL positive based on cytosolic staining in minimum of 10 % tumor cells. Human testis tissue was used as the positive control for TKTL1 and a negative control, for which the primary antibody was substituted with the same concentration of mouse IgG.

### Bisulfite treatment of DNA and quantitative methylation-specific PCR (MS-qPCR)

Genomic DNA was extracted using phenol chloroform (Qiagen), and bisulfite modification of genomic DNA was performed according to the manufacturer’s instructions (Qiagen). Methylation specific and methylation independent primers (Sigma Aldrich) were used to quantify the relative levels of methylated and unmethylated products within PCR amplified samples. The primers are as follows: *TKTL1* methylated primer (forward) 5'-GAC GTC TAA AAA ACG AAT AAC GC-3' and (reverse) 5'-AAA GAA CAT TTT GTA TTC GCG C -3'; *TKTL1* unmethylated primer (forward) 5'-CAA CAT CTA AAA AAC AAA TAA CAC C-3' and (reverse) 5'-GAA AGA AGA TTT TGT ATT TGT GTG G-3'; *β-actin* primer (forward) 5'-TGG TGA TGG AGG AGG TTT AGT AAG T -3' and (reverse) 5'-AAC CAA TAA AAC CTA CTC CTC CCT TAA -3'.

### Data set analysis

We acquired data form The Cancer Genome Atlas (TCGA) data portal (www.cbioportal.org). We started our analysis with the Level 3 data download (normalized data) of Illumina Infinium Human Methylation 450 k BeadChip array (450 k array) (http://www.illumina.com/products/methylation_450_beadchip_kits.html), and Illumina Hiseq RNA-Seq Platforms with RNA-seq gene and RNA-seq exon expression datasets (http://www.illumina.com/applications/sequencing/rna.html) of melanoma from the TCGA website (https://tcga-data.nci.nih.gov/tcga/dataAccessMatrix.htm?mode=ApplyFilter&showMatrix=true&diseaseType=SKCM&tumorNormal=TN&tumorNormal=T&tumorNormal=NT). Methylation measurement of each CpG probe was called β and was calculated as the methylated signal divided by the sum of the methylated and the unmethylated signal. Two methylation probes in accordance with Illumina annotation and UCSC genome browser that overlapped with our targeted region by MS-QPCR were selected. As there were some CpG sites between the two probes that was not covered by 450 k platform, we only investigated the correlation between these two probes in the 450 k platform by Spearman correlation coefficients statistical test (*p* < 0.05) to estimate methylation level of our targeted region. Subsequently, correlations between the two probes were separately assessed with *TKTL1* gene expression and exon expression. All analysis was performed by R statistical programming language (3.1.1).

### 5-aza-2′-deoxycytidine (5aza) treatment

Melanoma cell line LM-MEL-44 was treated with 0.1 μM of 5aza (Sigma) for 4 days. The medium and the 5aza were refreshed on alternate days. Cells were confluent at time of harvest.

### *TKTL1* overexpression construct and RNA interference transfection

The plasmid encoding *TKTL1* (clone ID 4825931) was obtained from Life Technologies and the *TKTL1* gene was subcloned into pcDNA3.1(+) vector (Promega). Cells were transfected using Lipofectamine 2000 (Life Technologies). Empty vector control used was pcDNA3.1(+). For transient siRNA transfection, cells at 30 % confluence were transfected using a control siRNA and two different Silencer select siRNAs targeting *TKTL1* (s224894 and s15774) at 10nM final concentration (Ambion) with Lipofectamine RNAiMAX according to the manufacturer’s protocol (Invitrogen).

### Immunoblot analysis

Cells were lysed in RIPA buffer (Sigma) and protein concentration quantified using the BCA protein assay (Thermo Scientific). Samples were separated using NuPAGE 4–10 % BisTris gels (Life Technologies) and MES [2-(*N*-morpholino) ethanesulfonic acid] SDS running buffer (Life Technologies). Transfer to PVDF membrane (Milipore) used a semi-dry transferblot (Bio-Rad Laboratories). Blocking was performed with Odyssey blocking buffer (Millenium Science), followed by incubation with anti-TKTL1 antibody (mouse monoclonal antibody, clone JFC12T10, 1:100) or anti-GAPDH antibody 1:1000 (Cell Signalling) overnight at 4 °C, and IRDye 680/800 secondary goat anti-mouse and anti-rabbit antibodies (Millenium Science) were incubated for one hour at room temperature. Images were acquired using Odyssey infrared imaging system (LI-COR Biosciences).

### Immunofluorescence

LM-MEL-44 transfected with TKTL1 overexpression plasmid construct was incubated for 72 h and then fixed with 4 % paraformaldehyde, stained with anti-TKTL1 rabbit polyclonal antibody (ab87187, Abcam) which was applied at 3 μg/mL concentration overnight at 4 °C and with Alexa flour 555 (anti-rabbit) conjugated secondary antibody for 45 min at room temperature (Molecular probes, USA). Cells were counter stained with DAPI for 10 min.

### Measurement of extracellular glucose and lactate

Cell-free supernatant of cultured melanoma cells was collected, and glucose and lactate concentrations were determined using colorimetric glucose and lactate assay kits (Biopharma). The assays were performed according to the manufacturer’s instructions and absorbance was measured at 340 nm.

### Cytofluorometric analysis

Cell viability and cell cycle analysis was assessed after staining with propidium iodide. *TKTL1* was over-expressed or depleted in melanoma cells for 48 h. Cells were stained with 200 μl propidium iodide solution consisting of 50 μg/ml propidium iodide, 0.1 mg/ml RNAse A and 0.05 % Triton-X (all Sigma) in PBS. Cells were incubated overnight at 4 °C and analyzed by flow cytometry using the FL2 channel.

### Proliferation assay

MTS colorimetric assay was performed using standard protocols. Twenty thousand cells were seeded per well of a 96 well plate and treated as indicated. Relative cell numbers were measured using the CellTiter 96® AQueous One Solution Cell Proliferation Assay (Promega Corporation).

### Invasion assay

Invasion assays were performed with Boyden chamber insert (6.5 mm diameter inserts with 8.0 μm pores) coated with Matrigel (Becton, Dickinson and Company) as described previously [70]. Briefly, melanoma cells were seeded at 40,000 cells per insert. 600 μl of media containing 10 % FCS was added as chemo attractant to the lower compartment. The cells were incubated overnight at 37 °C and subsequently fixed with 4 % paraformaldehyde, stained with 0.1 % Crystal Violet and cells on the upper surface of the insert were removed. Invaded cells on the bottom side were counted from three random fields of view observed with a 10X objective lens. Average of invaded cells across three fields of view was calculated from independent experiments repeated three times.

### Oxythiamine treatment

Melanoma cells were treated with varying dose of oxythiamine (Sigma) as indicated for 24, 48 and 72 h. Following treatment cells were subjected to proliferation, invasion and cytofluorometric analyses.

### Statistical analysis

All statistical comparisons of data sets were performed using Student’s two-tailed t-test in Prism software version 5.00 (GraphPad Software Inc). Statistical significance was set at *p* <0.05.

## Additional files

Additional file 1: Figure S1.
*TKTL1* is expressed in a subset of melanoma tumours and melanoma cell lines. (A) Representative staining patterns for TKTL1 in metastatic melanoma tumors are shown. Arrows indicate nuclear staining for Melanin. Original magnification, 200 μm. (B) QRT-PCR for expression level of *TKTL1* in a panel of 53 metastatic melanoma cell lines. (C) High magnification staining pattern of TKTL1 in melanoma cell line LM-MEL-59. Arrows indicate nuclear staining for Melanin. Original magnification, 200 μm. (PPTX 1827 kb) Additional file 2: Figure S2. Correlation of *TKTL1* gene expression with DNA methylation in melanoma patients. Exon expression data was compared to methylation patterns of TKTL1 and Spearman correlation coefficients were calculated. Methylation status at the CpG site (A) cg09892236 and (B) cg23106779 was inversely correlated with *TKTL1* gene expression in melanoma samples. (PPTX 114 kb) Additional file 3: Figure S3.
*TKT* and *TKTL2* expression in melanoma cells. (A) *TKT*, (B) *TKTL1* and (C) *TKTL2* expression in LM-MEL-59 after treatment with two *TKTL1 siRNAs* or control siRNA for 72 h. Testis tissue was used as positive control for the expression of *TKT*, *TKTL1* and *TKTL2*. (PPTX 69 kb) Additional file 4: Figure S4. TKTL1 expression in melanoma cells after gain and loss of function experiments. (A) Western Blotting with the mouse monoclonal anti-TKTL1 antibody showed reduction in TKTL1 levels after siRNA treatment in LM-MEL-59 after 72 h. GAPDH was used a loading control. (B) Western Blotting of TKTL1 confirmed expression of TKTL1 after transfection of a *TKTL1* expression vector in LM-MEL-44. Blots were probed with GAPDH as a control for loading and transfer. (C) Immunofluorescence confirmed increase in TKTL1 localization in LM-MEL-44 after transfection with TKTL1 expression vector. In contrast no TKTL1 was detected in LM-MEL-44 transfected with empty control vector. Scale bar =100 μm). (PPTX 590 kb) Additional file 5: Figure S5. TKTL1 expression in melanoma affects proliferation of cells. (A) Measurement of absorbance of LM-MEL-59 cells after *TKTL1* siRNA or control siRNA treatment for 48 h by performing MTS assay. (B) Measurement of absorbance of LM-MEL-44 cells after overexpression of *TKTL1* or empty vector for 48 h. (PPTX 57 kb) Additional file 6: Figure S6. Oxythiamine treatment in melanoma cells. Measurement of absorbance of (A) LM-MEL-59 or (B) LM-MEL-44 cells after treatment with 10 μM, 100 μM Oxythiamine or control media for 24, 48 and 72 h by performing MTS assay. (C) Cell cycle phases were determined by propidium iodide staining of LM-MEL-59 cells and subsequent flow cytometric analysis of percentage of cells in subG0, G0-G1, S and G2-M phases after treatment of LM-MEL-59 with 100 μM Oxythiamine or control media. Values are ± SD of three experiments. (D) Representative images of invasion of LM-MEL-59 is shown after treatment with 100 μM Oxythiamine (scale bar = 50 μm) and (E) invasion was quantified. (PPTX 183 kb)

## Abbreviations

5aza: 5-aza-2’-deoxycytidine
^18^FDG-PET: Fluorodeoxy glucose positron emission tomography
CTAg: Cancer Testis Antigens
MAGE-A: Melanoma Antigen Family –A
MS-qPCR: Quantitative Methylation-Specific PCR
qRT-PCR: Quantitative Reverse Rranscriptase-PCR
SKCM: Skin Cutaneous Melanoma
TCGA: The Cancer Genome Atlas
TKT: Transketolase
TKTL1: Transketolase-like 1
TKTL2: Transketolase-like 2
TMA: Tissue Microarray
TSS: Transcription Start Site
