# Supplementary figures and images for: Transketolase-like 1 ectopic expression is associated with DNA hypomethylation and induces the Warburg effect in melanoma cells
Source: BMC Cancer. 2016 Feb 22;16:134. doi: 10.1186/s12885-016-2185-5 (PMC4763451; doi:10.1186/s12885-016-2185-5)

## Slide 1
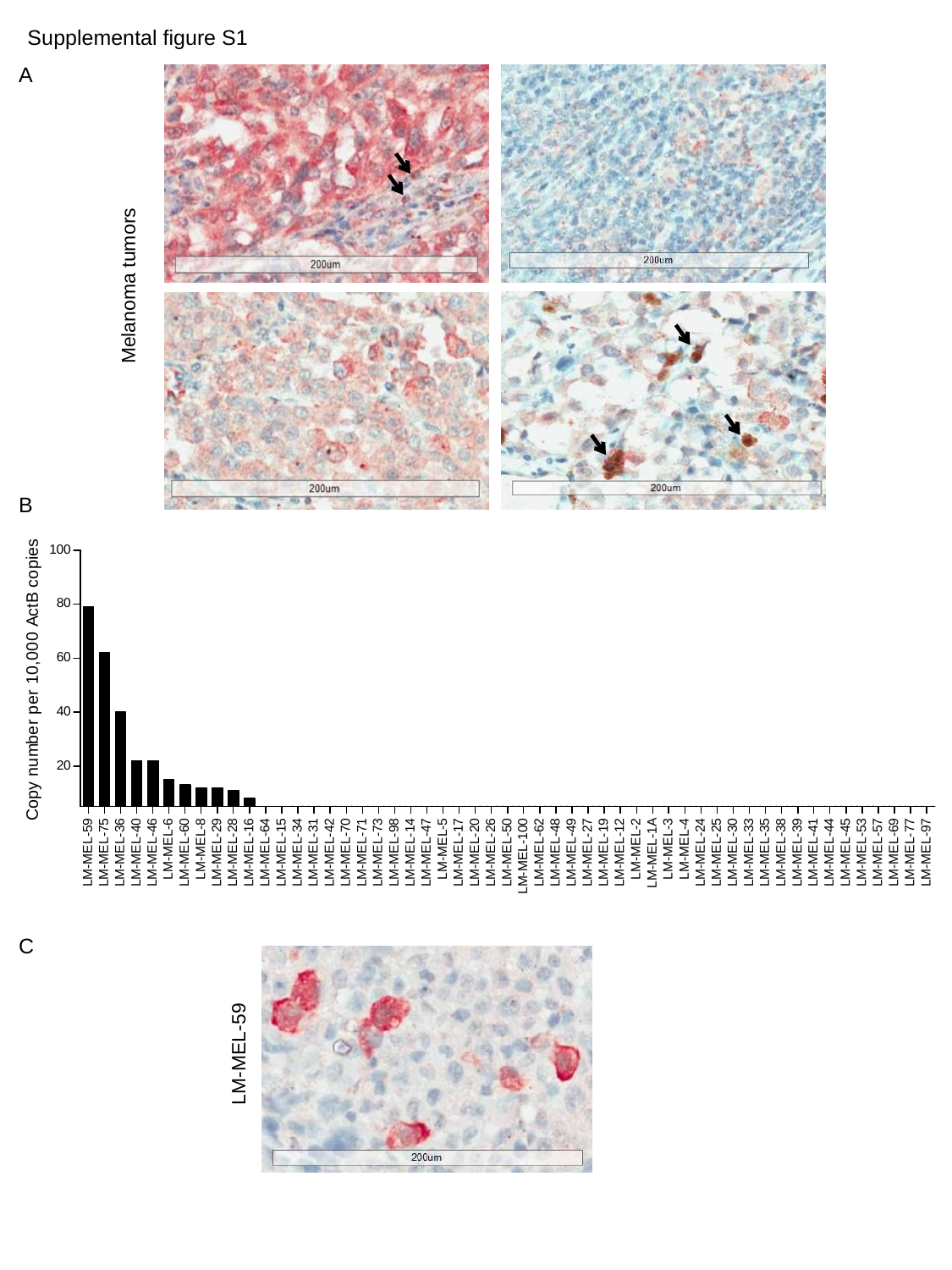

Supplemental figure S1
A
Melanoma tumors
B
C
LM-MEL-59

Supplement: Additional file 1: Figure S1. — TKTL1 is expressed in a subset of melanoma tumours and melanoma cell lines. (A) Representative staining patterns for TKTL1 in metastatic melanoma tumors are shown. Arrows indicate nuclear staining for Melanin. Original magnification, 200 μm. (B) QRT-PCR for expression level of TKTL1 in a panel of 53 metastatic melanoma cell lines. (C) High magnification staining pattern of TKTL1 in melanoma cell line LM-MEL-59. Arrows indicate nuclear staining for Melanin. Original magnification, 200 μm. (PPTX 1827 kb) [file 12885_2016_2185_MOESM1_ESM.pptx]

## Slide 1
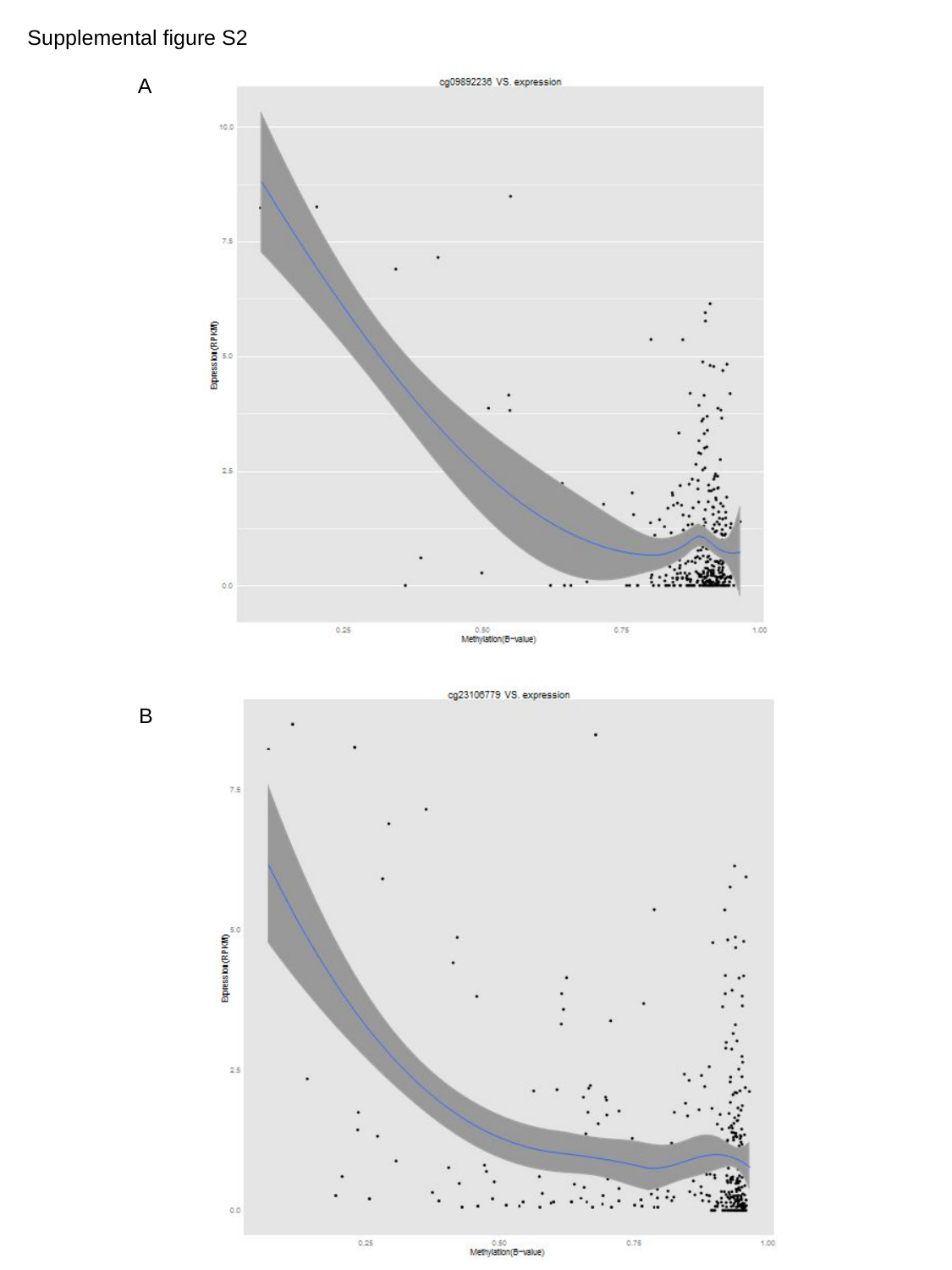

Supplemental figure S2
A
B

Supplement: Additional file 2: Figure S2. — Correlation of TKTL1 gene expression with DNA methylation in melanoma patients. Exon expression data was compared to methylation patterns of TKTL1 and Spearman correlation coefficients were calculated. Methylation status at the CpG site (A) cg09892236 and (B) cg23106779 was inversely correlated with TKTL1 gene expression in melanoma samples. (PPTX 114 kb) [file 12885_2016_2185_MOESM2_ESM.pptx]

## Slide 1
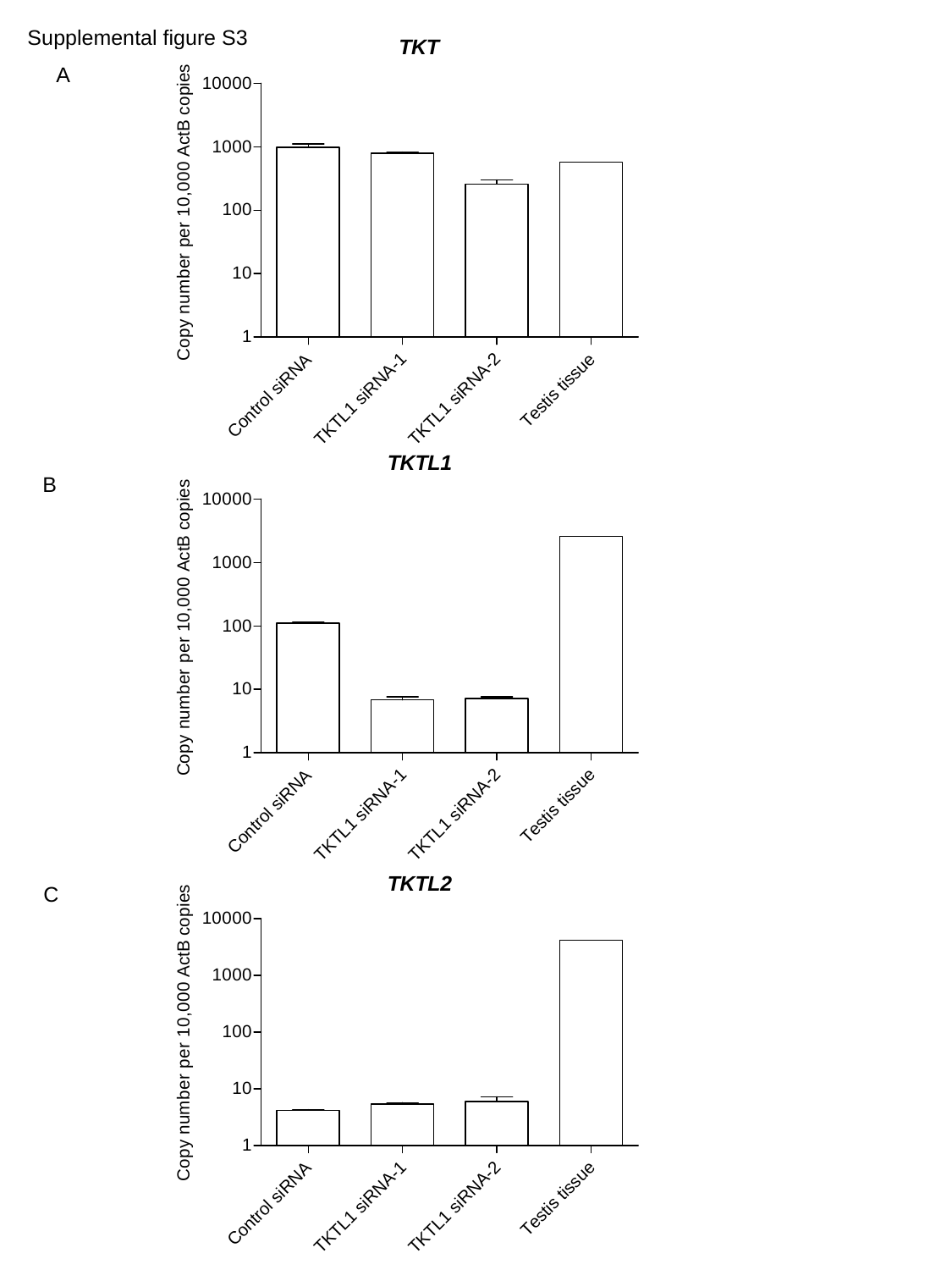

Supplemental figure S3
A
B
C

Supplement: Additional file 3: Figure S3. — TKT and TKTL2 expression in melanoma cells. (A) TKT, (B) TKTL1 and (C) TKTL2 expression in LM-MEL-59 after treatment with two TKTL1 siRNAs or control siRNA for 72 h. Testis tissue was used as positive control for the expression of TKT, TKTL1 and TKTL2. (PPTX 69 kb) [file 12885_2016_2185_MOESM3_ESM.pptx]

## Slide 1
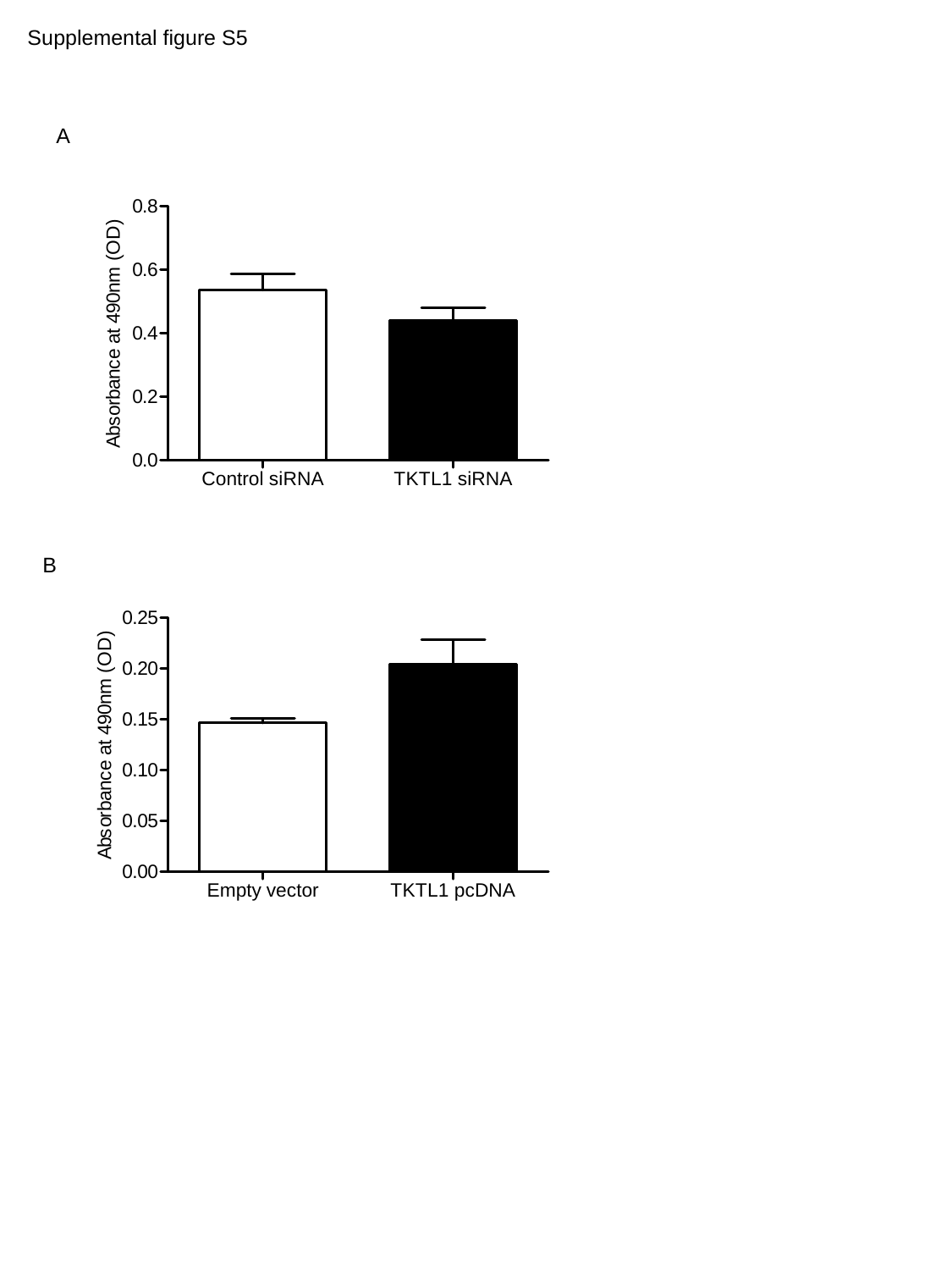

Supplemental figure S5
A
B

Supplement: Additional file 5: Figure S5. — TKTL1 expression in melanoma affects proliferation of cells. (A) Measurement of absorbance of LM-MEL-59 cells after TKTL1 siRNA or control siRNA treatment for 48 h by performing MTS assay. (B) Measurement of absorbance of LM-MEL-44 cells after overexpression of TKTL1 or empty vector for 48 h. (PPTX 57 kb) [file 12885_2016_2185_MOESM5_ESM.pptx]

## Slide 1
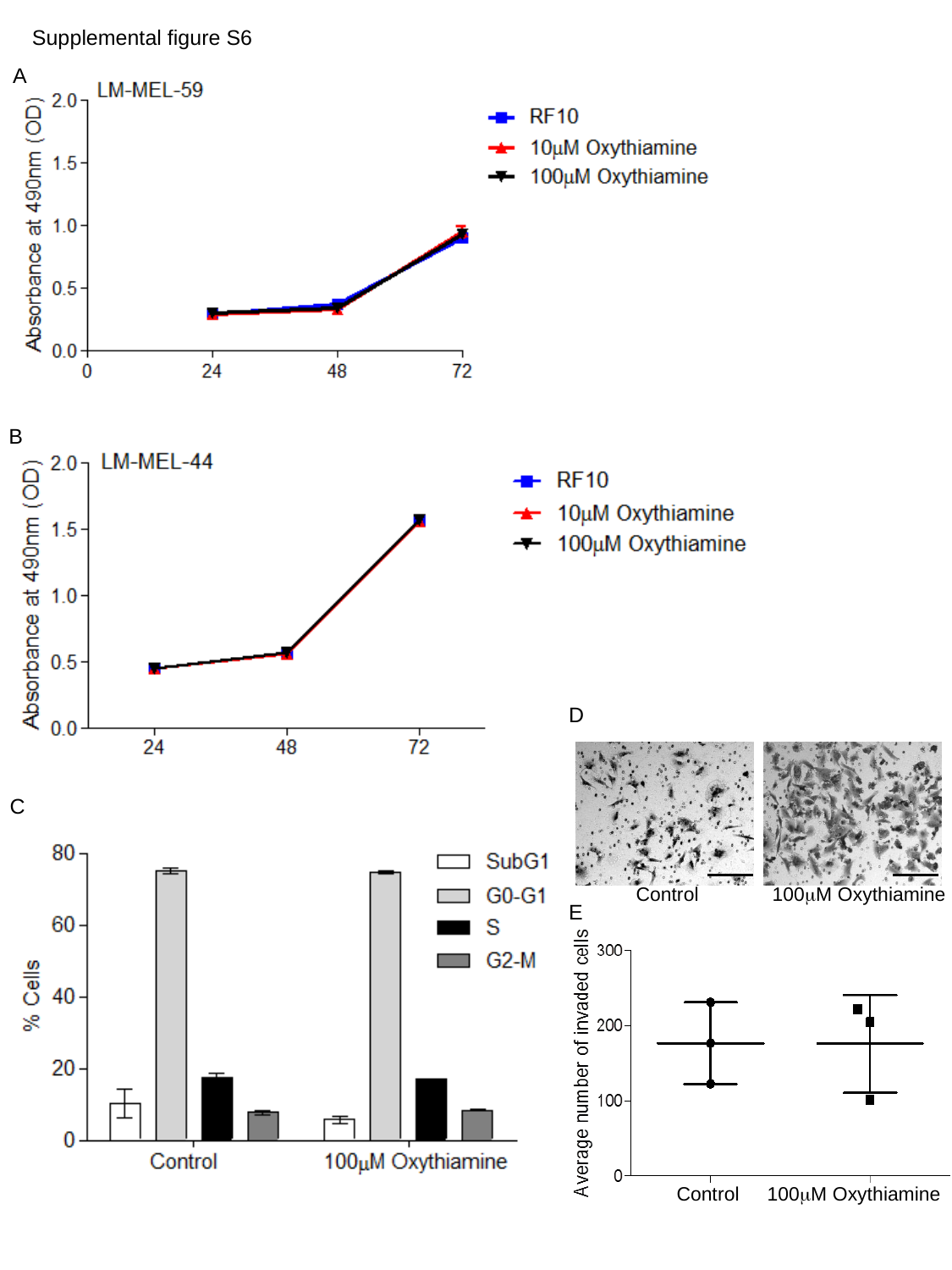

Supplemental figure S6
A
B
D
C
Control
100μM Oxythiamine
E
Control
100μM Oxythiamine

Supplement: Additional file 6: Figure S6. — Oxythiamine treatment in melanoma cells. Measurement of absorbance of (A) LM-MEL-59 or (B) LM-MEL-44 cells after treatment with 10 μM, 100 μM Oxythiamine or control media for 24, 48 and 72 h by performing MTS assay. (C) Cell cycle phases were determined by propidium iodide staining of LM-MEL-59 cells and subsequent flow cytometric analysis of percentage of cells in subG0, G0-G1, S and G2-M phases after treatment of LM-MEL-59 with 100 μM Oxythiamine or control media. Values are ± SD of three experiments. (D) Representative images of invasion of LM-MEL-59 is shown after treatment with 100 μM Oxythiamine (scale bar = 50 μm) and (E) invasion was quantified. (PPTX 183 kb) [file 12885_2016_2185_MOESM6_ESM.pptx]
