# Supplementary material for: Transketolase-like 1 ectopic expression is associated with DNA hypomethylation and induces the Warburg effect in melanoma cells
Source: BMC Cancer. 2016 Feb 22;16:134. doi: 10.1186/s12885-016-2185-5 (PMC4763451; doi:10.1186/s12885-016-2185-5)
Supplement: Additional file 4: Figure S4. — TKTL1 expression in melanoma cells after gain and loss of function experiments. (A) Western Blotting with the mouse monoclonal anti-TKTL1 antibody showed reduction in TKTL1 levels after siRNA treatment in LM-MEL-59 after 72 h. GAPDH was used a loading control. (B) Western Blotting of TKTL1 confirmed expression of TKTL1 after transfection of a TKTL1 expression vector in LM-MEL-44. Blots were probed with GAPDH as a control for loading and transfer. (C) Immunofluorescence confirmed increase in TKTL1 localization in LM-MEL-44 after transfection with TKTL1 expression vector. In contrast no TKTL1 was detected in LM-MEL-44 transfected with empty control vector. Scale bar =100 μm). (PPTX 590 kb) [file 12885_2016_2185_MOESM4_ESM.pptx]

## Slide 1
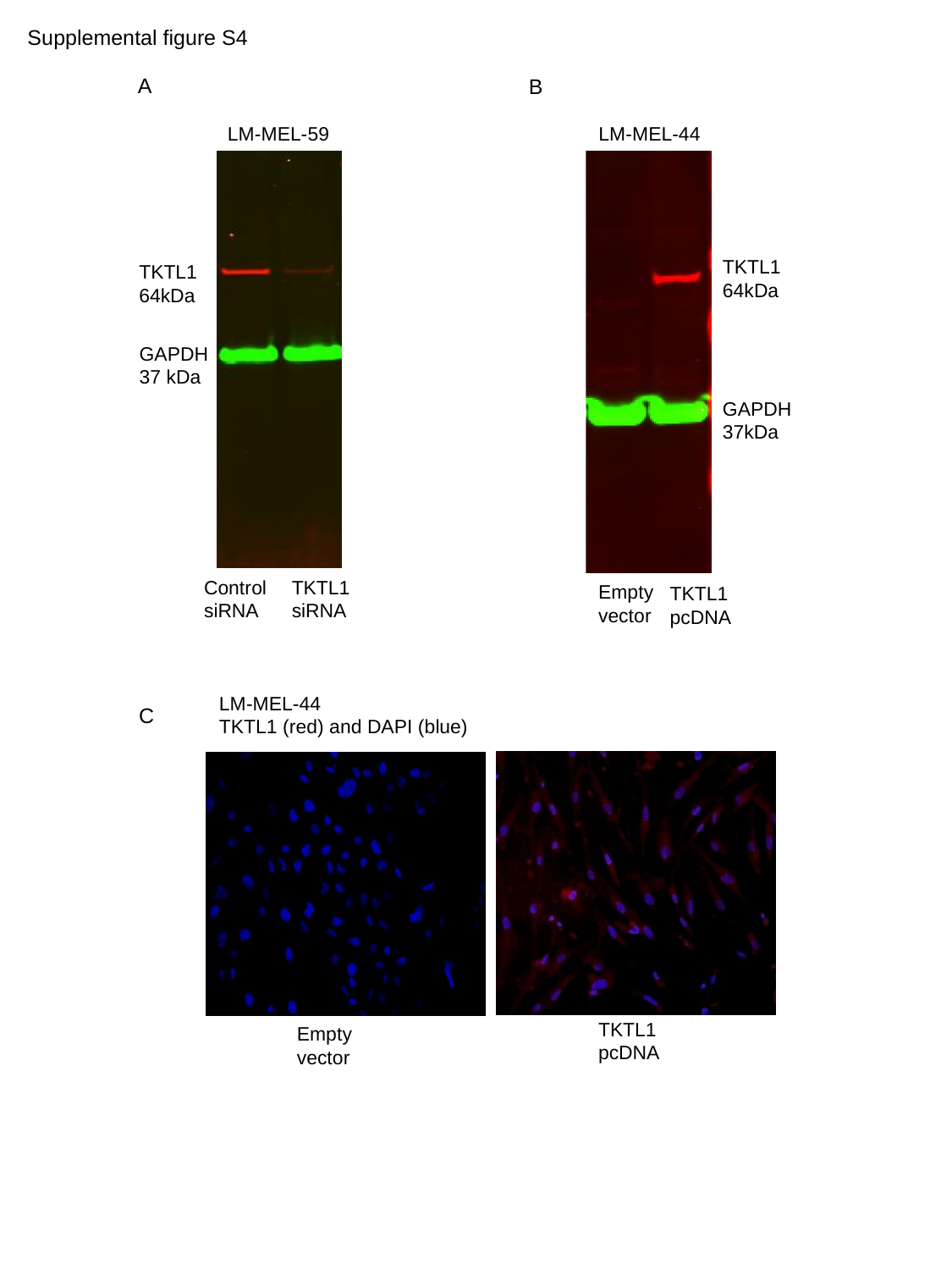

Supplemental figure S4
A
B
LM-MEL-59
LM-MEL-44
TKTL1
64kDa
TKTL1
64kDa
GAPDH
37 kDa
GAPDH
37kDa
Control
siRNA
TKTL1
siRNA
Empty
vector
TKTL1 pcDNA
LM-MEL-44
TKTL1 (red) and DAPI (blue)
C
TKTL1 pcDNA
Empty
vector
